# Supplementary material for: Live Biotherapeutic Lactococcus lactis GEN3013 Enhances Antitumor Efficacy of Cancer Treatment via Modulation of Cancer Progression and Immune System
Source: Cancers (Basel). 2022 Aug 23;14(17):4083. doi: 10.3390/cancers14174083 (PMC9455052; doi:10.3390/cancers14174083)
Supplement: Supplementary file 1 [file cancers-14-04083-s001.zip › cancers-1829782-supplementary.pdf]

cancers

MDPI

Supplementary Materials

Live Biotherapeutic *Lactococcus lactis* GEN3013 Enhances Antitumor Efficacy of Cancer Treatment via Modulation of Cancer Progression and Immune System

Sujeong Kim [2,†], Yunjae Kim [2,†], Suro Lee [1], Yulha Kim [1], Byungkwan Jeon [1], Hyerim Kim [1,*] and Hansoo Park [1,2,*]

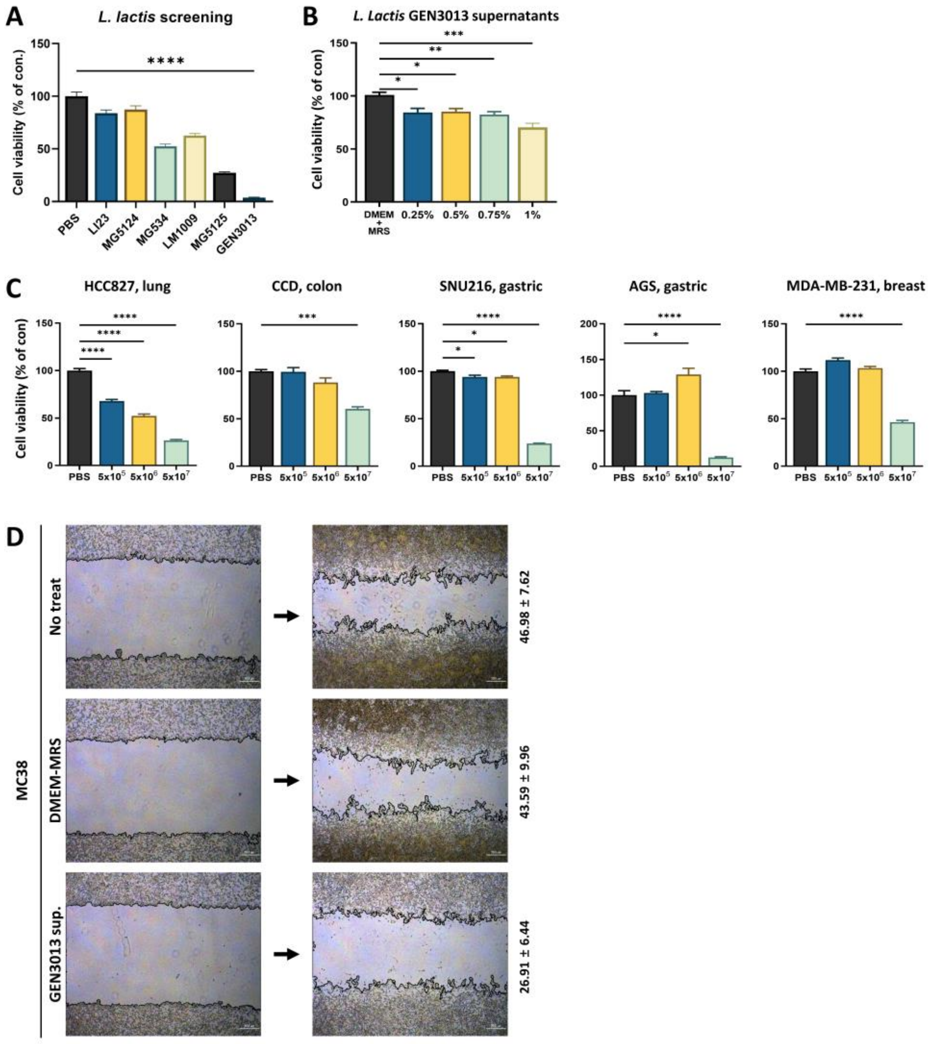

Supplementary Figure S1. Direct effects of *Lactococcus lactis* GEN3013 on cancer cell lines. (A) The lysates of several *Lactococcus lactis* strains were treated on MC38 cancer cell lines. The cell viability was measured by MTT assay and calculated relative to PBS control. (B) Various percent concentrations of *L. lactis* GEN3013 supernatants were treated on MC38 cells dose-dependently and incubated for 72 h. The cell viability was measured by WST-8 assay and calculated relative to MRS+DMEM control. (C) Various human cancer cell lines were treated with 0.5% *L. lactis* GEN3013 lysates in dose dependent manner and incubated for 72 h. The cell viability was measured by MTT assay and calculated relative to PBS control. (D) When treating *L. lactis* GEN3013 supernatants (26.91% ± 6.44%) to MC38 cancer cells, it also decreased the mobility of the cancer cells compared to DMEM-MRS mixture control (43.59% ± 9.96%) or no treat control (46.98% ± 7.62%).

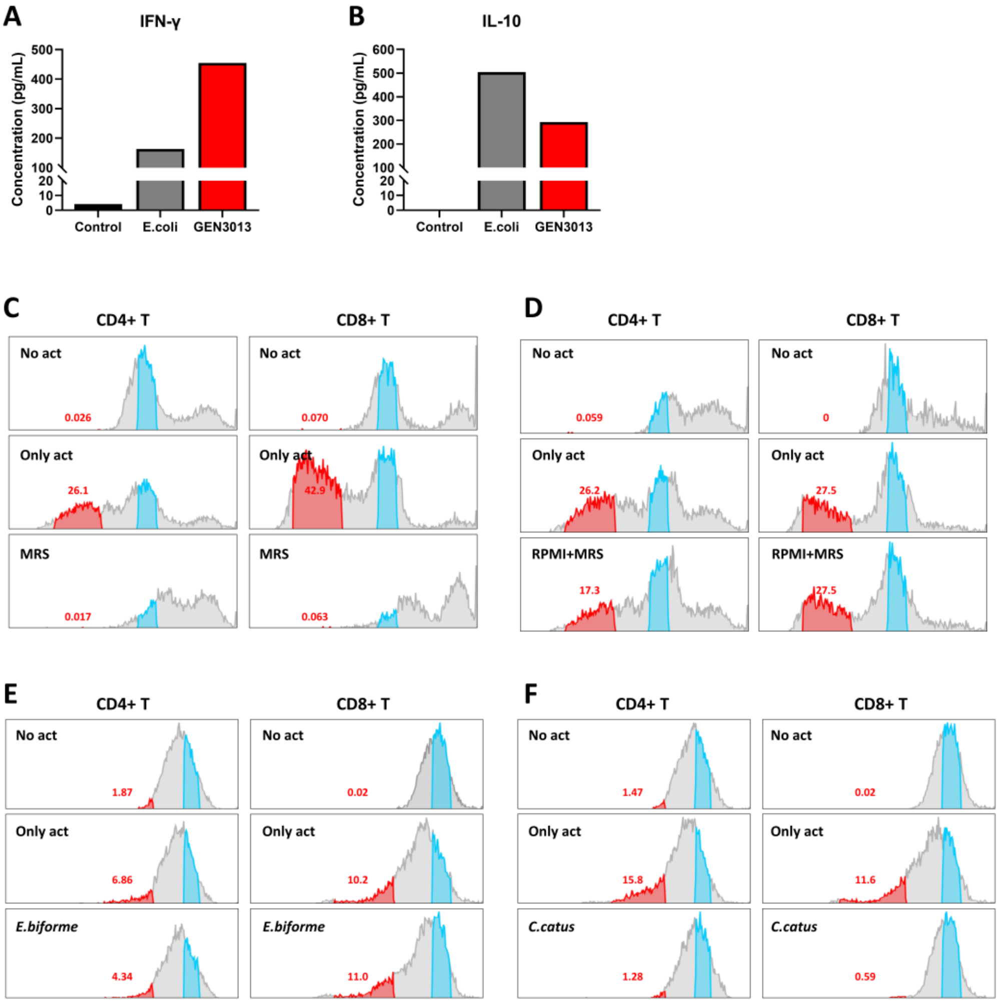

Supplementary Figure S2. Immune-boosting effects of *Lactococcus lactis* GEN3013. Human T cell activation was observed by ELISA of IFN-gamma (A) and IL-10 (B) with or without *L. lactis* GEN3013 treatment. Preliminary T cell assay was conducted with two controls, (C) MRS media (bacteria culture media) and (D) a mixture of MRS and RPMI (the culture medium for T cells). Two additional species, which are known not to harm humans, were confirmed by T cell proliferation assay. (E) *E. biforme* had no significant effect T cell proliferation and (F) *C. catus* inhibited the T cell proliferation.

Supplementary Table S1. Conditions for metabolic analysis of *Lactococcus lactis* GEN3013-derived metabolites

	Molecular Weight	Theoretical *m/z*	Adducts	Retention Time (min)	GEN3013 (1)	GEN3013 (2)	GEN3013 (3)	GEN3013 (4)	GEN3013 (5)
Vaccenic acid	282.256	283.2631568	[M+H]+	30.260	6.886	6.753	6.825	6.833	6.874
S-Adenosylmethionine	398.137	399.1445153	[M+H]+	0.810	6.927	6.636	6.799	6.699	6.733
Oleic acid	282.256	299.2591686	[M-H+H2O]-	30.230	5.005	4.908	4.963	5.040	5.154
N,N-Dimethylformamide	73.053	145.0982512	[2M-H]-	0.650	5.093	5.152	5.382	5.107	5.298
Methylthioadenosine	297.090	298.0968368	[M+H]+	5.950	7.371	7.235	7.351	7.320	7.249
Indole lactic acid	205.074	188.070605	[M+H-H2O]+	1.660	7.781	7.289	7.428	7.331	7.382
GABA (gamma-aminobutyric acid)	103.063	102.0560521	[M-H]-	0.680	5.661	5.394	5.418	5.239	5.415
Citrate	192.027	191.0197261	[M-H]-	0.600	6.747	6.296	6.329	6.327	6.518
Betaine	117.079	116.0717021	[M-H]-	0.700	6.193	5.666	5.770	5.610	5.772

Normalized log10, 5 technical replicates
